# Are high medical costs incurred by people with disabilities excessive?: An empirical analysis of Korean National Health Insurance Data

**DOI:** 10.1371/journal.pone.0262653

**Published:** 2022-01-20

**Authors:** Min Jung Hong, Changwoo Lee, Clara Lee, Ye-Soon Kim, Jae Yeon Jeong, Seeun Park, Dong Wook Shin, Euichul Shin

**Affiliations:** 1 Gimpo Woori Hospital, Gimpo, Gyeongi-do, Republic of Korea; 2 Institute of Health Policy and Management, Medical Research Center, Seoul National University, Seoul, Republic of Korea; 3 Department of Thoracic and Cardiovascular Surgery, Asan Medical Center, Ulsan University School of Medicine, Seoul, Republic of Korea; 4 Department of Healthcare and Public Health Research, National Rehabilitation Center, Seoul, Republic of Korea; 5 Department of Health Administration, Yonsei University Graduate School, Wonju, Gangwon-do, Republic of Korea; 6 School of Nursing, University of Wisconsin-Madison, Madison, Wisconsin, United States of America; 7 Department of Family Medicine, Samsung Medical Center, Sungkyunkwan University School of Medicine, Seoul, Republic of Korea; 8 Department of Preventive Medicine, College of Medicine, The Catholic University of Korea, Seoul, Republic of Korea; University of Oxford, UNITED KINGDOM

## Abstract

A crude comparison of medical costs between people with disabilities (PWD) and without disabilities (PWoD) shows a much higher expenditure among PWD and such results have been a cause for further stigmatization. This study aims to empirically analyze whether the medical costs for PWD are actually high when characteristics related to medical costs are adjusted. Ten percent of the total population was randomly selected from the Korean National Health Insurance (NHI) Database in 2016. A crude comparative analysis was performed to calculate the medical cost of PWD and PWoD. A subsequent multiple regression analysis was conducted to adjust factors affecting the medical costs such as socioeconomic status, disease, and health behavior-related characteristics. The medical cost for PWD was 3.6 times higher than that for PWoD by crude comparison. However, after multiple regression analysis, margin of difference decreased to 1.5 times although the cost for PWD remained higher. Substantial decrease in higher medical costs for PWD after multiple analyses compared to crude analysis implies that additional adjustment using variables such as disease severity, not available in the NHI database, may predict a further reduction in differences. Thus, it is difficult to determine that the medical expenditure for PWD is excessive.

## Introduction

The number of people with disabilities (PWD) continues to grow in Korea due to the increased prevalence of chronic disease and population aging [1]. The prevalence of chronic diseases such as hypertension and diabetes is 31.2% and 12.4%, respectively [2], and Korea has become an aged society in which the elderly account for over 14% of the total population in 2017 [3]. As of 2018, the number of PWD in Korea was approximately 2.58 million, accounting for about 5% of the total population [4, 5]. As the number of PWD in Korea increased and health and medical care expenditure for them have also increased, Korean society perceived disability to be a political issue [6]. In order to improve the vulnerable health status of PWD, the Korean government implemented the “Act on Guarantee of Right to Health and Access to Medical Services for Persons with Disabilities” in 2017 [7]. Recently the announcement of the 5th Comprehensive Policy Plan for People with Disabilities (2018–2022) established a support system for PWD that details comprehensive services that are tailored to the medical and welfare needs of PWD [8]. In addition, for the continuous provision of medical services aimed at improving the quality of life of PWD and management of chronic disease, a dedicated primary care system and health examination institutions for PWD have been introduced [9].

However, the policy for improving the vulnerable health status of PWD is likely to increase the social cost burden [8]. Contributing factors to medical care expenditure increase in PWD include the higher incidence of preventable disease due to physical and financial barriers to timely access of curative and preventative services. The duration of treatment periods is also relatively longer, resulting in increased severity of disease and disability and a higher tendency for complications [6, 10, 11]. In addition, a higher proportion of older people is a causative factor for higher medical care expenditure in PWD [12].

Statistical results of medical care expenditure of PWD show that the annual medical care cost of PWD per capita was about 4,264 USD, which was 3.3 times higher than the total medical care cost per capita per year of 1,292 USD [13, 14]. The total medical care cost for PWD was approximately 9,915,014 USD, which was 15.6% of the total medical care cost (63,650,850 USD) for the total population of Korea (Ministry of Health and Welfare and the National Rehabilitation Center in 2016), which, when taking into consideration the population composition, reflects substantially high medical expenses. Since the statistics have been released, various media in South Korea simultaneously published articles expressing serious concerns that the medical care costs for PWD are excessive by highlighting the fact that about 5% of the total population takes up too much of the total medical care costs. However, such a crude analysis and press reports have contributed to forming distorted public opinions that PWD are unnecessarily excessive in medical care utilization, which may lead to social conflict ultimately. Therefore, an in-depth analysis adjusting the different characteristics between PWD and people without disabilities (PWoD) is imperative when comparing the two groups [15, 16].

In this study, to compare the medical care costs between PWD and PWoD, data were adjusted using factors that could affect medical expenditure, including socioeconomic characteristics such as sex and age, as well as disease and health behavior characteristics using the National Health Insurance (NHI) database. By doing so, this study attempted to empirically analyze the medical care cost solely influenced by disability.

## Methods

### Data

This study uses the 2016 Korean National Health Insurance Database, which refers to national health insurance information that includes membership qualifications and insurance premiums, medical service utilization, and health check-up results of all nationals, which are collected, maintained, and managed by the National Health Insurance Service (NHIS). The NHIS provides the database for policy development and academic research purposes, and it is fully anonymized for the protection of patient’s personal information. The NHIS stipulates legal restrictions to sharing the data publicly or using other purposes than the research.

**Fig 1:**
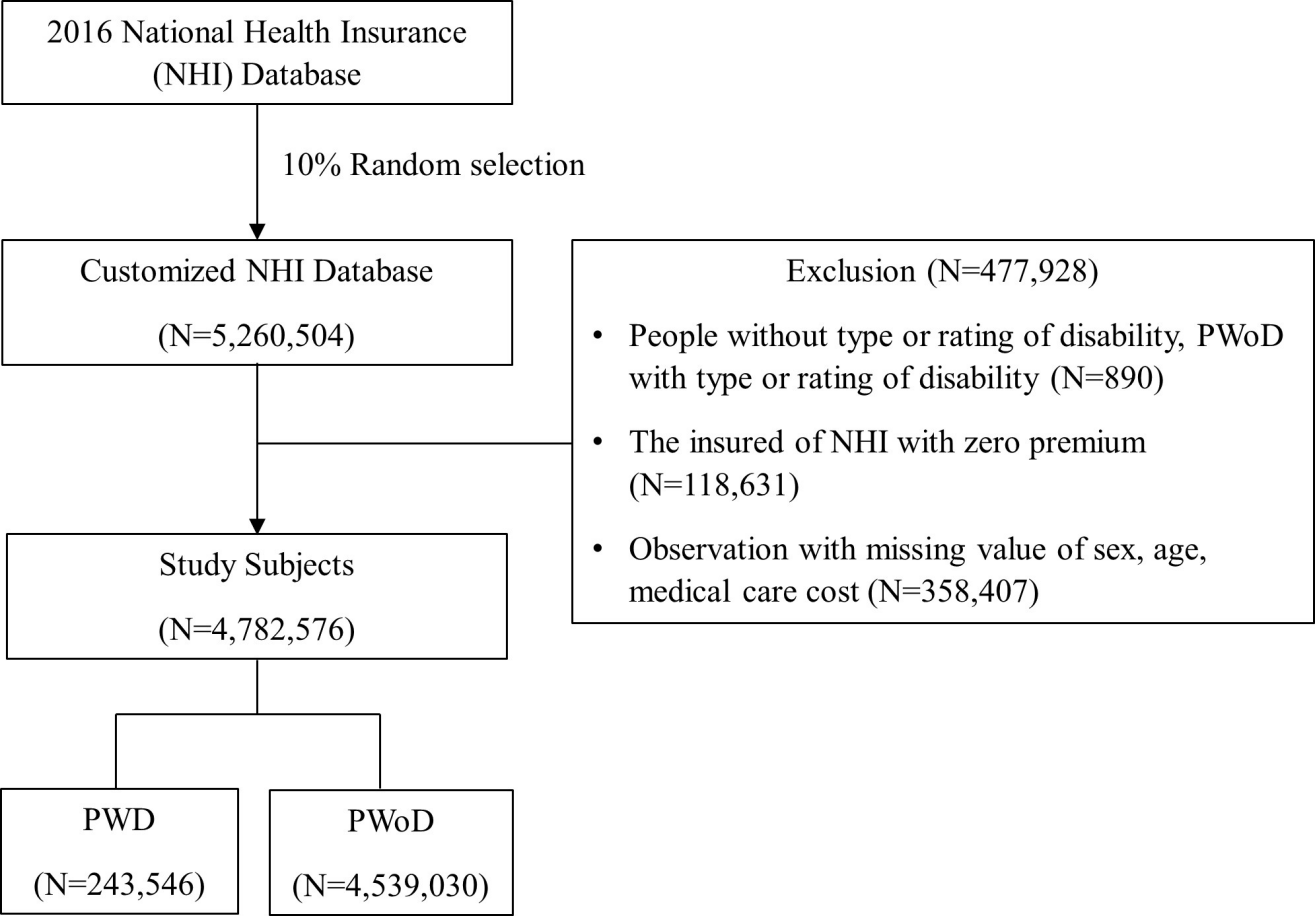
Study subject selection process.

The study subjects were randomly selected from 10% of the total population from the 2016 database (N = 5,260,504). Of these, observations were removed on implementation of data cleaning rules and 243,546 PWD and 4,539,030 PWoD were selected for a total of 4,782,576 persons; PWD without type or rating of disability, PWoD with type or rating of disability (N = 890), insured with zero premium(N = 118,631), missing values of sex, age, and medical care cost (N = 358,407). The conditions of observations with zero premium are as follows. 1) benefit status change from insured of National Health Insurance to recipients of Medical Aid or vice versa or 2) missing value of insurance premium variable due to error. It was unable to discern the observations with zero premium due to errors, and the variable ‘income level’ was generated based on insurance premium level; thus, all observations with zero premium mentioned above were excluded (Fig 1).

### Variables

The total annual medical expenditure for the year 2016 was chosen as the dependent variable. In this study, medical expenditure refers to the direct medical care cost incurred when patients use medical services in medical institutions, which is the sum of the amount of money paid by the NHIS and out-of-pocket money paid by the patients.

The analysis variable of interest for this study is the dummy variable indicating whether an individual has a disability or not. The current “Act on Welfare of Persons with Disabilities” requires that PWD be registered within the government registry for PWD according to the administrative procedure of the Ministry of Health and Welfare. Disability-related information is available in the NHI database. Types of disability are classified into external disorders (physical, brain lesion, visual, hearing, language, and facial), internal disorders (renal, cardiac, respiratory, hepatic, ostomy and urostomy, and epilepsy), and mental disorders (autism, intellectual, and mental).

The socio-demographic characteristics included gender, age, residence, type of health insurance, and income level. Ages were grouped into 20-year intervals of under 19 years, 20–39, 40–59, 60–79, and over 80 years. Based on the urbanization of residential areas, Seoul and six metropolitan cities are classified as metropolitan cities, the other cities as medium-sized cities, and the remainder as rural areas. The types of medical coverage are categorized into employees, self-employed, and medical aid. For income level, the health insurance premium is used as an index variable for income. The deciles of health insurance premiums are categorized into quintiles 1 to 5 in the lower order.

Disease and health-related characteristics include the status of chronic disease, smoking, high-risk drinking, and physical activity such as walking. Chronic disease status was defined based on 12 chronic diseases outlined by the “Standard Guide for Statistics Calculation of Disease and Services” published by the Health Insurance Review and Assessment Service using International Classification of Disease codes. These are hypertension (I10-I13, I15), diabetes (E10-E14), heart disease (I05-I09, I20-I27, I30-I52), cerebrovascular disease (I60-I69), malignant neoplasm (C00-C97, D00-D09, D32-D33, D37-D48), liver disease (B18-B19, K70-K77), mental and behavioral disorders (F00-F99, G40-G41), respiratory tuberculosis (A15-A16, A19), nervous system disease (G00-G37, G43-G83), thyroid disorders (E00-E07), chronic renal failure (N18), and arthritis (M00-M09, M11-M19, M45) [17]. A patient with at least one chronic disease as main or as sub-diagnoses is defined as a chronic disease patient. Variables for the characteristics of health behaviors were retrieved using information from the general health check-up questionnaire survey that is routinely administered to those who receive the general health check-up periodically advised by the NHIS. Those who answered ‘yes’ to the current smoking questionnaire were defined as smokers. Otherwise, current non-smokers, including ex-smokers, are regarded as non-smokers. High-risk drinkers were classified as those who drank more than five glasses of beer for men and four glasses for women three times a week. Physical activity was defined as walking for 30 minutes or more per day in total, for at least three times a week.

### Statistical analysis

Descriptive statistics, t-tests, and chi-square tests were conducted to examine the distribution and differences in socio-demographic characteristics, disease and health characteristics, and medical care costs for PWD and PWoD. Medical care costs skewed to the left in the distribution are log-transformed. Multiple linear regression analysis was performed to analyze the effects of disability on medical care costs while controlling for sociodemographic, disease, and health behavior characteristics. Stata version 15.0, statistical software was used for all analyses, and statistical significance was set at α = 0.05.

This research was conducted with approval (MC19ZESE0111) after being reviewed by the Institutional Review Board, The Catholic University of Korea.

## Results

### General characteristics of the study subjects

Table 1 shows the distribution of characteristics of the study subjects. The proportion of PWD that were male was 57.7%, which was higher than the percentage of 48.3% seen in PWoD and was statistically significant. The mean age of PWD was 60.1 which was higher than the mean age of 39.8 years in PWoD, and was statistically significant. The proportion of those aged 60 and over in PWoD was just 20.0%, which was statistically lower than the proportion seen in PWD (57.4%).

**Table 1:** General characteristics of the study subjects (N = 4,782,081).

Variable		PWD		PWoD		*p*-value
		N	(%)	N	(%)	
**Socio-demographic characteristics**						
Sex*	Male	140,526	(57.7)	2.192,351	(48.3)	<0.001
	Female	103,020	(42.3)	2,346,667	(51.7)	
Age group*	≤19	8,110	(3.3)	930,501	(20.5)	<0.001
	20–39	21,383	(8.8)	1,235,978	(27.2)	
	40–59	74,282	(30.5)	1,466,107	(32.3)	
	60–79	106,673	(43.8)	721,706	(15.9)	
	≥80	33,098	(13.6)	184,739	(4.1)	
	Mean ± SD	60.1 ± 17.8		39.8 ± 21.6		<0.001
Urbanicity of Residence*	Metropolitan city	86,124	(35.4)	1,790,739	(39.5)	<0.001
	Medium-sized city	114,273	(47.0)	2,175,750	(47.9)	
	Rural area	42,903	(17.6)	572,292	(12.6)	
Type of health Insurance*	Employee	136,142	(55.9)	3,172,782	(69.9)	<0.001
	Self-employed	63,566	(26.1)	1,225,538	(27.0)	
	Medical aid	43,838	(18.0)	140,710	(3.1)	
Income level*	Q1 (low)	88,894	(36.5)	903,267	(19.9)	<0.001
	Q2	36,532	(15.0)	907,806	(20.0)	
	Q3	36,775	(15.1)	916,884	(20.2)	
	Q4	50,170	(20.6)	1,166,531	(25.7)	
	Q5 (high)	31,174	(12.8)	653,620	(14.4)	
**Disease and health behavior related characteristics**						
Chronic disease	Yes	204,092	(83.8)	2,078,876	(45.8)	<0.001
	No	39,454	(16.2)	2,460,154	(54.2)	
High-risk drinking*§	Yes	10,817	(15.5)	276,940	(22.2)	<0.001
	No	59,186	(84.5)	969,835	(77.8)	
Smoking*§	Yes	13,750	(19.6)	267,320	(21.4)	<0.001
	No	56,294	(80.4)	980,047	(78.6)	
Physical activity*§	Yes	20,161	(28.8)	342,964	(27.5)	<0.001
	No	49,843	(71.2)	904,178	(72.5)	
**Total**		243,546	(100.0)	4,539,030	(100.0)	

PWD, people with disabilities; PWoD, people without disabilities; SD, standard deviation; Q, quintile

*, statistically significant at α = 0.05

§, denominator only with observation of the respondents.

Analysis according to distribution by income level showed that 25.7% of PWoD were in the fourth quintile (highest income level) and 19.9% in the first quintile (lowest income level), showing a lower proportion of PWoD in the lower income quintile. In contrast, 36.5% of PWD were in the first quintile with more distribution in lower-income levels compared to PWoD, showing a statistically significant difference between the two groups. Furthermore, the proportion of PWD in the collective low-income quintiles Q1 and Q2 was 51.5%, which was higher than that seen in PWoDs (39.9%). The proportion of high-income quintiles Q4 and Q5 for PWD was 33.4%, which was lower than that for PWoDs (40.1%). All results were statistically significant.

Analysis of the characteristics of disease and health behavior showed that the prevalence of chronic disease was 83.8% for PWD, approximately twice as high as the 45.8% in PWoD. In the case of health behavior, the proportion was measured based on individuals who had no missing values in the questionnaire. 15.5% of the PWD and 22.2% of the PWoD were high-risk drinkers. The proportion of smokers was 19.6% in the PWD group and 21.4% in the PWoD group, showing that the proportion of high-risk drinking and smoking for PWoD was higher than that for PWD. The proportion of physical activity(walking) was 28.8% for PWD and 27.5% for PWoD, indicating a slightly higher proportion of physical activity in PWD. All results were statistically significant.

### Medical care costs of PWD and PWoD

A crude (unadjusted) analysis of the total annual cost of treatment for PWD and PWoD per person shows that the average cost for PWD is 3,917 USD and that for PWoD is 1,080 USD, indicating that PWD spend approximately 3.6 times more than PWoD. There was a statistically significant difference in the average medical care costs between the two groups (Table 2). The total annual medical care cost per person for 15 types of disabilities shows that the costs for renal disorders (19,933 USD), and hepatic disorders (11,783 USD) are the top two conditions with high-cost expenditure, followed by brain lesions (7,268 USD), respiratory disorders (5,711 USD), and cardiac lesions (5,590 USD). The renal disorder type of disability with the highest cost spending is about five times higher than the average medical care cost per person for PWD (Table 3).

**Table 2:** Average medical care cost of the study subjects by disability status (USD).

Disability status	Mean ± SD	N	Ratio (a/b)	*p*-value
PWD	3,917.41 ± 8,216.00	243,546	3.63	<0.001
PWoD	1,080.48 ± 2,977.17	4,539,030		

PWD, people with disabilities; PWoD, people without disabilities; SD, standard deviation.

**Table 3:** Average medical care cost per person by types of disability (USD).

Type of disability	Mean±SD	N	(%)
**Disability of external physical function**			
Physical disability	2,954.17±6,536.35	124,581	(51.11)
Brain lesion	7,268.18±11,471.23	24,954	(10.25)
Visual impairment	2,802.39±5,288.92	24,207	(9.94)
Hearing impairment	3,178.11±5,621.57	24,310	(9.98)
Language disorder	4,063.92±18,826.47	1,797	(0.74)
Facial disorder	2,149.09±4,007.90	263	(0.11)
**Disability of internal organs function**			
Renal disorder	19,933.03±14,896.65	7,299	(3.00)
Cardiac lesion	5,590.12±9,383.77	1,094	(0.45)
Respiratory disorder	5,710.81±8,800.00	1,240	(0.51)
Hepatic disorder	11,782.60±10,796.29	1,046	(0.43)
Intestinal and urinary tract disorder	4,962.92±7,352.35	1,386	(0.57)
Epilepsy disorder	3,341.28±4,903.85	997	(0.41)
**Mental function**			
Autistic disorder	1,026.24±2,360.92	1,995	(0.82)
Intellectual disability	1,800.59±5,163.00	17,517	(7.25)
Mental disorder	2,956.47±5,717.24	10,559	(4.38)
**PWD**	3,917.41±8,216.00	243,546	(100.0)

SD, standard deviation; PWD, people with disabilities.

### Impact of disability on medical care cost: Multiple linear regression analysis

Multiple regression analysis was conducted to examine the effects of disability on medical care costs, controlling for related characteristics. The results show that the medical care cost of PWD was 1.5 times higher than that of PWoD. The F test for the overall model was statistically significant at the 5% level, and the explanatory power was approximately 30.1% (Table 4).

**Table 4:** Effect of disability on medical care cost per person: Multiple linear regression analysis.

Variable		Coef.	Exp(coef)	p-value
Disability*	No	-	-	
	Yes	0.399	1.490	<0.001
**Adjustment variable**				
Sex*	Male	-	-	
	Female	0.162	1.176	<0.001
Age group*	≤19	‒0.598	0.550	<0.001
	20–39	‒0.421	0.656	<0.001
	40–59	0.276	1.318	<0.001
	60–79	0.656	1.927	<0.001
	≥80	-	-	
Region*	Metropolitan city	-	-	
	Medium-sized city	‒0.011	0.989	<0.001
	Rural area	‒0.003	0.997	0.079
Type of health Insurance*	Employee	-	-	
	Self-employed	0.067	1.069	<0.001
	Medical aid	0.260	1.297	<0.001
Income level*	Q1 (low)	-	-	
	Q2	0.021	1.022	<0.001
	Q3	0.099	1.104	<0.001
	Q4	0.093	1.097	<0.001
	Q5 (high)	0.063	1.065	<0.001
Chronic disease*	No	-	-	
	Yes	1.404	4.072	<0.001
High-risk drinking*	No	-	-	
	Yes	‒0.036	0.965	<0.001
Smoking*	No	-	-	
	Yes	‒0.086	0.918	<0.001
Physical activity*	Yes	-	-	
	No	‒0.053	0.948	<0.001
Constant		12.479		<0.001

F = 253269.307; Adj R^2^ = 0.301; -, Reference

*, Statistically significant at α = 0.05; Q, quintile.

## Discussion

As shown in the study, the total annual medical cost per person for PWD was found to be 3,917 USD, which is 3.6 times higher than that of PWoD, at 1,080 USD. This result is similar to the study results conducted by Kim et al. using the NHI database, which shows that the average medical cost per capita in 2015 for PWD was 3,922 USD and 974 USD for PWoD, indicating that PWD spends four times more than PWoD [18]. This finding is also similar to the study results conducted by the National Rehabilitation Center, which shows a 3.3 times difference in the total medical care cost per capita between PWD (4,240 USD) and the total population (1,292 USD) as of 2016 [13]. Although the comparative group for the study is the entire population, not PWoD, it is considered to be similar to the results of this study, given that the proportion of PWD is about 5% of the total population. However, an in-depth analysis is needed to determine whether such higher medical expenditures are actually excessive [16].

In this study, multiple regression analyses were performed to adjust relevant characteristics affecting medical care costs, including socio-economic characteristics, disease status, and health behavior-related characteristics available from the NHI database. As a result, the ratio of annual average medical care cost for PWD to PWoD substantially decreased from 3.6 times to 1.5 times (Table 4). This result suggests that the medical care cost of PWD is overestimated compared to that of PWoD if the medical care costs of PWD and PWoD are simply compared without considering factors affecting medical care costs, such as socio-demographic, disease status, and health behavior-related characteristics.

If multiple regression analyses are performed using variables known to have a significant impact on medical care costs, but are not available on the NHI database (for example, disease severity), the impact of disability on the cost of medical care might be further reduced, resulting in a similar or even lower cost for PWD than PWoD. Therefore, considering all these factors, it is difficult to conclude that the medical expenditure of PWD is higher than that of PWoD based on a crude comparative analysis of medical costs. Furthermore, existing government policies are insufficient to support the medical costs incurred by PWD. The annual per-capita out-of-pocket medical care cost for PWD was approximately 7,993 USD, while that for PWoD was about 2,469 USD in 2017 [16], which is almost three times the difference. It is hoped that the results of this study will be used as an opportunity to develop and provide more diverse and practical economic support measures for medical care usage by PWD and not discourage the use of needed medical services.

Suspecting that many of observed differences in characteristics (Table 4) might actually be confounded by the age differences between PWD and PWoD. As PWD are older on average, then they are probably less likely to be employed, more likely to be retired, and thus have lower income. However, as a result of in-depth analysis by income decile to confirm this, the differences in medical expenses between PWD and PWoD are not changed significantly as they are older on average. People are less likely to be employed, more likely to be retired, and have lower income as they get older applies to both PWD and PWoD.

This study also showed considerable variations in average medical care costs among the types of disabilities. The type of disability with the highest average medical care cost per capita was renal impairment, and its costs were 19,933 USD, and the lowest was autistic disorders (1,026 USD). The difference in medical care costs between the two is about 19.4 times, showing a significant difference. Therefore, when developing a healthcare support policy related to PWD medical care costs, disability types and medical utilization patterns should be considered.

The limitations of this study are as follows. First, the medical care costs analyzed in the study were calculated based on the expenses within insurance benefits. Thus, it does not include out-of-pocket money, which limits the accurate calculation of the actual medical care costs for PWD. In addition, all summarized characteristics show statistically significant differences between groups (p<0.001) in multiple regression analysis. This could be due to the very large sample size, very small differences are showing as significant, rather than there being meaningful differences in the values. This should be considered when interpreting the analysis results.

One of the strengths of this study is that the comparison group for the disabled (PWD) was those without disability and PWoD, not the entire population. Previous studies have selected the entire population as a comparative group of the disabled [13, 14]. Therefore, this study might contribute to the literature related to PWD by using an appropriate comparator group for the PWD target group. In addition, random sampling of 10% of the nation’s population in the design of the study subject provided further added value.

## Conclusion

Various statistical data books show that medical care costs of PWD are much higher than those of the general population and are perceived as being excessive. In this study, multiple regression analyses were performed to adjust for sociodemographic factors, disease status, and characteristics between PWD and PWoD using the NHIS database. As a result, the medical care cost of PWD decreased substantially compared to that of PWoD, from 3.6 times to 1.5 times. This implies that additional adjustment using major variables such as disease severity, which is not available in the NHI database, may predict a further reduction in differences in medical care costs between PWD and PWoD. Thus, it is difficult to determine that the medical expenditure incurred by PWD is excessive through a mere crude (unadjusted) comparative analysis.

## References

[1] Ministry of Health and Welfare. Korea Institute for Health and Social Affairs. 2017 National Survey on Persons with Disabilities. Sejong: Ministry of Health and Welfare; 2017:31–33.

[2] Ministry of Health and Welfare. Korea Health Statistics of The 7th Korea National Health and Nutrition Examination Survey. Sejong: Ministry of Health and Welfare; 2017:224,231.

[3] Statistics Korea. Rate of aging population statistics 2017 [Internet]. Daejeon: Statistics Korea. 2017 [cited 2021 Apr 8]. Available from: https://kosis.kr/statHtml/statHtml.do?orgId=101&tblId=DT_1YL20631&conn_path=I2.

[4] Statistics Korea. 2018 Census [Internet]. Daejeon: Statistics Korea. 2019 [cited 2021 Apr 8]. Available from: http://kosis.kr.

[5] Ministry of Health and Welfare. 2018 Status of registration for the Disabled [Internet]. Ministry of Health and Welfare. 2018 [cited 2019 Sep 23] Available from: http://www.mohw.go.kr.

[6] Korea Disabled People’s Development Institute. 2017 The Disabled White Book. 2017:29–40,118–131.

[7] Act on guarantee of right to health and access to medical services for persons with disabilities, Act No.13661. 2015 [cited 2019 Sep 23]. Available from: https://elaw.klri.re.kr.

[8] Ministry of Health and Welfare. The 5th Comprehensive Policy Plan for Persons with Disabilities. Sejong: Ministry of Health and Welfare; 2018.

[9] Ministry of Health and Welfare, National Rehabilitation Center. 2018 Health Care for the Disabled. Seoul: National Rehabilitation Center; 2019:2–6.

[10] Kim JY, Kang MW, Seo WY. Research on chronic diseases and health behaviors of persons with disabilities in Korea. Seoul: Korea Disabled People’s Development Institute; 2019:10:30–33.

[11] Dejong G, Palsbo SE, Beatty PW, Jones GC, Knoll T, Neri MT. The organization and financing of health services for persons with disabilities. Milbank Q. 2002;80(2):261–301. doi: 10.1111/1468-0009.t01-1-00004 12101873 PMC2690107

[12] Kim Y, Lee JY, Lee BS, Kim WO, Lee JS, Moon NJ, et al. Analysis of utilization of medical care benefit and improvement plan for medical security. Seoul: National Healthcare Insurance Service; 2005:55–58.

[13] Ministry of Health National Rehabilitation Center. 2016 Disability and Health Statistics. Seoul: National Rehabilitation Center; 2018:430–441.

[14] National Health Insurance Service, Health Insurance Review & Assessment service. 2016 National Health Insurance Statistical Yearbook. Seoul: National Health Insurance Service; 2017:168–183.

[15] Moon JH, Kim YS. Comparison of Socio-economic Characteristics, Presence of Chronic Disease, Medical use and Expenditure between Olders with and without Disabilities: Using Data of Korea Health Panel. Korean Aging Friendly Industry Association 2018;10(2):14.

[16] National Rehabilitation Center, Korea. A Conference Proceedings on Health Statistics for the Disabled: Health of the Disabled: How is it changing? [Internet]. National Rehabilitation, Korea. 2021 [cited 2021 April 8]. Available from: https://www.nrc.go.kr:2451/viewer/skin/doc.html?fn=20210317130534034229_1.pdf&rs=/viewer/result/202104/.

[17] Health Insurance Review & Assessment service. Standard guide for statistics calculation of disease and services. Wonju: Health Insurance Review & Assessment service; 2016:64–65.

[18] Kim SH, Lim SJ, Moon SW, Choi EH, Lee JH. Model development of health care management doctor for severely disabled persons. Wonju: National Healthcare Insurance Service; 2016:142–151.

